# Comparing income inequalities in healthcare utilization in the low income community in suburban Kuala Lumpur and Malaysia

**DOI:** 10.1186/1472-6963-14-S2-P144

**Published:** 2014-07-07

**Authors:** Nithiah Thangiah, Hazreen Abdul Majid, Tin Tin Su

**Affiliations:** 1 Centre for Population Health (CePH), Department of Social and Preventive Medicine, Faculty of Medicine, University of Malaya, Kuala Lumpur, Malaysia

## Background

This paper aims to measure and compare income inequalities in healthcare utilization in the low income community in suburban Kuala Lumpur as compared to the national population level in Malaysia. The prevalence of those who sought outpatient treatment for acute and chronic illnesses and also prevalence of inpatient admission in both public and private health facilities were compared.

## Materials and methods

The data consists of 800 households with 3722 respondents from four Projek Perumahan Rakyat (PPR) among the low income community in suburban Kuala Lumpur and compared with data from the National Health and Morbidity Survey in 2006 consisting of 13,637 households with 56,710 respondents. The income inequalities are explored using concentration index (CI) with CI values below zero indicating pro-poor inequality whereas CI values above zero indicating pro-rich inequality.

## Results

For the low income community, analysis across ranked monthly household income reveals that in government-led public healthcare facilities, inequalities in outpatient attendances and inpatient admission to be in favor of the lower socioeconomic groups with CI values of -0.0874 and -0.0636 respectively (as compared to-0.1722 and -0.0869 at the national level). By contrast, in private healthcare facilities, inequalities in outpatient attendances and inpatient admission clearly to be in favor of the higher socioeconomic groups with significant CI values of 0.2090 and 0.2309 respectively (as compared to 0.1851 and 0.5176 at the national level).

## Conclusions

The low income community clearly has higher need for healthcare especially with rising prevalence of chronic illnesses and non-communicable diseases. As such, a pro-poor inequality is expected to exist in the utilization of healthcare in public healthcare facilities. Since similar patterns of utilization are observed both at the low income community level and national level, the lower socioeconomic groups are found to be benefitting through sufficient targeting of healthcare resources in Malaysia.

